# Editorial: Lymph node assessment in cervical cancer

**DOI:** 10.3389/fonc.2023.1324654

**Published:** 2023-11-01

**Authors:** Benedetta Guani, Enrique Chacon, Francesco Fanfani, Nicolò Bizzarri

**Affiliations:** ^1^ Service de Gynécologie, Hopital Cantonal hôpital fribourgeois (HFR) Fribourg, Fribourg, Switzerland; ^2^ Faculté de Sciences et Médecine, Université de Fribourg, Fribourg, Switzerland; ^3^ Department of Obstetrics and Gynecology, Clinica Universidad de Navarra, Pamplona, Spain; ^4^ Unità operativa complessa (UOC) Ginecologia Oncologica, Dipartimento di scienze della salute della donna, del bambino e di sanità pubblica, Fondazione Policlinico Universitario A. Gemelli, Istituti di ricovero e cura a carattere scientifico (IRCCS), Rome, Italy; ^5^ Università Cattolica del Sacro Cuore, Rome, Italy

**Keywords:** sentinel lymph node, lymphadenectomy, cervical cancer, lymph nodal metastases, lymph nodal involvement

The 2018 FIGO classification (1) of cervical cancer includes nodal involvement.

Lymph node status is the most important prognostic factor in early-stage cervical cancer. In fact, positive pelvic and para-aortic lymph nodes significantly impact disease-free survival and overall survival. Evaluation of lymph node status before surgery is important, as radical surgery is recommended for patients with negative lymph node metastasis (Zhou et al.), while positive lymph node status represents an indication for treatment with radio chemotherapy.

For this reason, the Research Topic was focused on lymph node assessment.

The two main topics of discussion were:

1. How to define the risk of lymph node invasion before surgery.
2. How to perform lymph node investigation via sentinel lymph node or lymphadenectomy.

## 1 Estimation of lymph node involvement risk

In this Research Topic, Peng et al. showed that lymph node metastasis is a significant independent predictor of recurrence.

The short-term and long-term outcomes of patients with lymph node metastasis before treatment are poor. For patients with lymph node metastasis before treatment, more active, individualized treatment strategies should be adopted.

Lymph node status is crucial to deciding whether patients with cervical cancer can be subjected to radical surgery in cases of apparent early-stage disease.

This point addresses the question of the frozen section, with the risk of not finding the low-volume metastases, versus the two-step strategy: first, sentinel lymph node (SLN) removal and analysis by ultrastaging, and second, radical surgery if nodes are free from metastases. This last strategy has to be counterbalanced with increasing costs and processing times in the case of negative nodes (N0), with twice as many hospitalizations and surgeries for patients. In addition, the second surgery, 10 days later, can be more difficult because of inflammation and postoperative adhesions (2).

However, the first strategy includes the risk of false negative sentinel lymph nodes. In the study by Balaya (3), the sensitivity and the negative predictive value of the frozen section were 42.3% and 89.7%, respectively. The international, multicenter, prospective, observational SENTIX trial (4) showed similar results: frozen sections failed to detect 54% of positive lymph nodes, including 28% of cases with macrometastases and 90% with micrometastases (5). In the recent systematic review and meta-analysis by Agustí (6), the pooled sensitivity of intraoperative sentinel lymph node frozen section analysis was 65% (95% CI, 51-77%) for macrometastases, micrometastases, and isolated tumor cells.

In this Research Topic, the authors proposed alternative strategies to assess the risk of lymph node invasion before surgery.

The CER-CAP application (Guani et al.) makes it possible to predict an individual risk of lymph node metastasis. With the CER-CAP application, the authors wanted to provide a practical tool for scheduling the surgical management of patients with early-stage cervical cancer.

Indeed, in the case of a high risk of nodal invasion according to the CER-CAP score, they proposed performing the lymph node evaluation first and waiting for the definitive results of ultrastaging before deciding on management. In the case of a low-risk score, the authors suggested proceeding directly to surgical treatment and avoiding the morbidity of a two-step procedure. If we consider only macrometastases, the prediction score applied to Senticol 1 and 2 is 100%, with no macrometastases detected in low-risk patients.

Another possibility is ncRNA analysis. Previous studies have analyzed the potential value of ncRNA expression in gynecological cancers [(7–13), Yang et al. (14–16)]. In the review presented in our Research Topic (Dabi et al.), the authors aimed to evaluate the contribution of ncRNAs in tissue and biofluid samples to determine lymph node status in cervical cancer, with potential implications for both surgical and adjuvant therapies. They supported the role of ncRNAs in physiopathology, differential diagnosis from normal tissue, and pre-invasive and invasive tumors. In biofluids, despite small studies, especially concerning miRNAs expression, promising data open new avenues to establish a non-invasive signature for lymph node status and a tool to predict response to neo- and adjuvant therapies, thus improving the management algorithm of patients with cervical cancer.

Other risk factors for recurrence are described in this special edition, like the pre-treatment C-reactive protein (CRP) levels (17).

CRP levels appear to be a reliable factor in determining cervical cancer prognoses (14–16).

In addition, based on the SEER database, Wang et al. constructed a quantitative and visual prognostic nomogram that predicted the prognosis of patients with lymph node metastases in cervical cancer to provide clinicians with a reference for diagnosis and treatment.

Recently, the potential application of a radiomics model to predict the risk of cervical cancer has been proposed with very interesting results (18, 19). The combination of radiomics with clinicopathologic markers may aid in predicting the risk of lymph node metastasis with high accuracy.

## 2 Pelvic lymphadenectomy for early-stage cervical cancer

Sentinel lymph node biopsy represents an alternative to pelvic lymphadenectomy for lymph node staging in early-stage cervical carcinoma.

In the 2023 update to the ESGO/ESTRO/ESP guidelines (20), SLN biopsy (without additional lymph node dissection) is recommended in T1A1 patients with positive lymph node space invasion (LVSI+) and T1A2 patients.

In the case of T1B1, T1B2, and T2A1 stages, if SLN is negative on the frozen section after a SLN biopsy, a systematic pelvic lymphadenectomy should be performed as the standard lymph node staging, according to the guidelines.

We expect a full pelvic lymphadenectomy to remove 15 nodes bilaterally, but the mean rate of node-positive patients in early-stage cervical cancer is less than 20%. This means that 80% of patients undergo unnecessary lymphadenectomy with potential complications without staging or therapeutic benefit.

In this special edition, Yong et al. sought to determine the minimum number of lymph nodes resected with lymphadenectomy that is associated with survival improvement.

A sufficient number of lymph nodes was associated with better long-term survival in FIGO stages IB-IIA. At least eight lymph nodes need to be examined for prognostic stratification. Excessive lymph node dissection (>17) may not confer an additional survival benefit.

A recent meta-analysis (21) showed that sentinel lymph node biopsy could be considered a standard surgical procedure (without lymphadenectomy) in patients with early-stage cervical cancer (T1A1 LVSI+ to T2A1) to reduce post-operative complications and improve the quality of life and prognosis.

Further prospective studies will be needed to confirm this hypothesis.

In conclusion, knowledge of lymph node status in cervical cancer is crucial to deciding the best treatment strategy for cervical cancer. A pre-operative tool to predict the risk of nodal metastasis, along with the use of sentinel lymph nodes, may lead gynecologic oncologists to obtain information about lymph nodes with reduced morbidity for the patients. The combination of innovative multi-omics approaches represents a future approach to understanding the risk of lymph node involvement in cervical cancer.

## Author contributions

BG: Conceptualization, Writing – original draft. EC: Validation, Writing – review & editing. FF: Supervision, Validation, Writing – review & editing. NB: Conceptualization, Supervision, Validation, Writing – review & editing.
